# Weight Status and Weight-Management Behaviors Among Philadelphia High School Students, 2007–2011

**DOI:** 10.5888/pcd10.130087

**Published:** 2013-09-26

**Authors:** Clare M. Lenhart, Katherine W. Bauer, Freda Patterson

**Affiliations:** Author Affiliations: Clare M. Lenhart, East Stroudsburg University, East Stroudsburg, Pennsylvania; Katherine W. Bauer, Temple University, Philadelphia, Pennsylvania. At the time of the study, Dr Lenhart was affiliated with Lehigh Valley Health Network, Allentown, Pennsylvania.

## Abstract

**Introduction:**

The prevalence of obesity among youth may be stabilizing and even declining in some areas of the United States. The objective of our study was to examine whether the stabilization in obesity prevalence among Philadelphia high school students was accompanied by changes in weight-management behaviors.

**Methods:**

We evaluated changes in self-reported weight status and weight-management behaviors by using data collected by the Youth Risk Behavior Survey in 2007, 2009, and 2011. We used multivariable regression models controlling for race/ethnicity and age to estimate prevalence.

**Results:**

Although the proportion of overweight and obese students did not change significantly during the study period, we found that approximately half of female students and 30% of male students reported trying to lose weight. Among female students, we observed significant increases in the proportion engaging in 5 or more days of physical activity per week (26.0% in 2007 to 31.9% in 2011; *P* = .003) and significant decreases in the proportion consuming at least 1 soda per day (31.1% in 2007 to 22.5% in 2011, *P* = .001). The proportion of female students who fasted for weight loss also increased significantly during the study period (12.2% in 2007 to 17.0% in 2011, *P* = .02). We found no significant changes in behavior among male students.

**Conclusion:**

Although the prevalence of obesity and overweight may have reached a plateau among Philadelphia high school students, most students still failed to meet recommendations for healthful weight-management behaviors. Continued public health initiatives are necessary to promote participation in healthful weight-management behaviors.

## Introduction

Recent data suggest that rates of childhood obesity in the United States are no longer rising (1). This stabilization has provided optimism that clinical and public health efforts to stem the childhood obesity epidemic have begun to take effect (2). Consistent with these national findings, recent data from Philadelphia showed declines in obesity rates among students in grades kindergarten through 12; rates among students in grades kindergarten through 8 and among male students declined significantly. Among students in grades 9 through 12, the prevalence of obesity declined, although not significantly, between 2006–2007 and 2009–2010 (3). The confluence of national attention to childhood obesity and programmatic and policy-based interventions in Philadelphia may account for these declines (3).

Although obesity rates among youth have reached a plateau or are decreasing among some populations, it remains important to identify the behavioral changes associated with these trends. For example, limiting the intake of sugar-sweetened beverages and time spent watching television are effective ways to help adolescents prevent excessive weight gain (4,5). It is also essential to consider whether the heightened focus on obesity in schools and communities across the United States is prompting adolescents to use weight-loss strategies that are harmful or counterproductive — such as fasting or using diet pills or laxatives — to healthful weight management (6).

The objective of our study was to examine whether the stabilization in obesity prevalence among Philadelphia high school students observed in a previous study (3) was accompanied by changes in weight-management behaviors. Study findings will offer insight into the weight-management behaviors of urban high school students, including those who want to lose weight. Findings may also illuminate whether the sharpened focus on childhood obesity has unintended consequences.

## Methods

We used data collected in Philadelphia by the Youth Risk Behavior Survey (YRBS). The YRBS is a surveillance system maintained by the Centers for Disease Control and Prevention to monitor youth health behaviors. The survey uses a 2-stage cluster sampling design to produce a representative sample of high school students; it is administered biennially. Standard YRBS procedures require passive parental consent, and nonopposing students are invited to anonymously complete the in-school survey. The survey is provided in a multiple-choice, self-administered, paper-and-pencil format (7). We used data collected for the Philadelphia YRBS in 2007 (N = 2,119), 2009 (N = 1,165) and 2011 (N = 1,239). In each of the data collection years, the school participation rate was 76% or more; within schools, of the students invited to complete the survey, the response rate ranged from 76% in 2007 to 78% in 2011.

We examined the following demographic variables: sex, age, and race/ethnicity. Body mass index (BMI) was calculated by using self-reported weight (kg) divided by the square of self-reported height (m); age- and sex-specific BMI percentiles were computed (8). Students whose BMI was at or above the 95th percentile were considered obese; students whose BMI was in the 85th through 94th percentile were considered overweight (9).

We also evaluated the following weight-related variables: weight perception, weight-loss intention, soda consumption (≥1 soda per day in past week), fruit and vegetable consumption (≥5 servings per day in past week), physical activity levels, screen time (television viewing and recreational computer use), and extreme weight-management strategies (Appendix). Because only 1.2% of students were classified as underweight during the study period, we classified underweight students as normal-weight students. Weight perception was measured by using a single item that asked students to self-identify as being one of the following: very underweight, slightly underweight, about the right weight, slightly overweight, or very overweight. Accurate weight perception was defined as being overweight or obese per BMI and self-identifying as overweight (either slightly or very) or as being of normal weight per BMI and self-identifying as normal weight. We classified as inaccurate all other combinations of BMI category and perceived weights status.

Physical activity levels were evaluated as a single item that asked students on how many days of the past 7 days they were active for at least 60 minutes; responses were dichotomized to 0 through 4 days and 5 or more days because 5 days or more of activity per week is recommended by the American College of Sports Medicine (10). Screen time was measured as average hours per day of television viewing and recreational computer use (combined); responses were dichotomized as 0 or 1 hour and 2 or more hours (11). Use of extreme weight-management strategies was measured by 3 yes/no questions on the use of fasting; diet pills, powders, or liquids; and vomiting in the past 30 days. Earlier versions of the YRBS have demonstrated appropriate validity and reliability (12,7).

We conducted statistical analyses on weighted data by using SAS, version 9.3, PROC SURVEY procedures (SAS Institute, Inc, Cary, North Carolina) to account for the complex sampling design of the YRBS. Sampling errors were estimated by using the primary sampling units and strata provided in the data and calculated through Taylor series linearization. Sampling weights were used to adjust for nonresponse and oversampling and to allow for generalizability of findings to the population of high school students in Philadelphia. We used descriptive statistics to examine the demographic characteristics of the samples. Individual multivariable regression models were developed to examine the prevalence of each of the study outcomes across the 3 study years. Regression models included students’ race/ethnicity and age as covariates to account for differences in these demographic characteristics across the study years. We used the *F* statistic to identify significant differences in the estimated prevalence rates across survey years and made post-hoc comparisons of prevalence rates for outcomes for which the overall *F* statistic indicated differences. Similar regression models and corresponding *F* statistics were developed to examine changes in weight-management behaviors among overweight and obese students who indicated a desire to lose weight.

## Results

The demographic composition of the sample was stable during the study period (Table 1). The proportion of female students ranged from 57% in 2007 to 51% in 2011. In 2007, an estimated 9% of respondents were aged 14 years or younger; 24% were aged 15, 28% were aged 16, 28% were aged 17, and 11% were aged 18 years or older. Approximately 54% were African American, 14% white, 8% Hispanic, 9% Asian, and 16% mixed race/ethnicity or other.

**Table 1:** Demographic Characteristics of Participating High School Students, Study on Weight Status and Weight-Management Behaviors, Philadelphia, 2007–2011

Characteristic	2007			2009			2011		
	Unweighted Count	Weighted Estimate (SD)	%	Unweighted Count	Weighted Estimate (SD)	%	Unweighted Count	Weighted Estimate (SD)	%
**Total**	2,119	39,867 (1,838)	—	1,165	37,982 (1,846)	—	1,239	34,509 (1,292)	—
**Sex**									
Female	1,194	22,875 (1,222)	57.4	653	19,840 (1,176)	52.2	669	17,645 (901)	51.1
Male	925	16,993 (1,115)	42.6	512	18,141 (1,302)	47.8	570	16,864 (974)	48.9
**Age, y**									
≤14	170	3,442 (570)	8.6	57	2,562 (471)	6.7	122	3,434 (637)	10.0
15	544	9,644 (1,215)	24.2	260	10,249 (1,255)	27.0	295	8,540 (1,037)	24.7
16	644	11,244 (1,001)	28.2	380	10,489 (973)	27.6	324	9,312 (941)	27.0
17	550	10,996 (1,225)	27.6	332	8,661 (892)	2.8	300	7,597 (845)	22.0
≥18	211	4,542 (734)	11.4	136	6,020 (1,461)	15.8	198	5,626 (816)	16.3
**Race/ethnicity**									
African American	1,191	214,690 (970)	53.9	643	24,868 (1,434)	65.5	648	20,430 (961)	59.2
Non-Hispanic white	291	5,480 (923)	13.7	143	4,825 (663)	12.7	164	5,080 (643)	14.7
Hispanic	138	3,206 (565)	8.0	98	2,249 (367)	5.9	99	2,407 (315)	7.0
Asian	184	3,377 (601)	8.5	87	1,663 (252)	4.4	122	1,948 (235)	5.6
Mixed race/other	315	6,336 (517)	15.9	194	4,377 (424)	11.5	206	4,636 (429)	13.4

We found no significant differences in the prevalence of obesity or overweight among female students from 2007 to 2011 (Table 2). In 2007, 31.1% of female students reported having at least 1 soda per day; this proportion decreased to 22.5% in 2011 (*P* = .001). Additionally, the prevalence of female students engaging in 5 or more days of physical activity in the previous week increased from 26.0% in 2007 to 31.9% in 2011 (*P* = .003). However, the prevalence of female students who reported fasting to lose weight also increased significantly from 12.2% in 2007 to 17.0% in 2011 (*P* = .02). We observed no other significant differences in weight-management behaviors or weight perceptions among female students.

**Table 2:** Self-Reported Weight Status and Weight-Management Behaviors Among Female and Male High School Students, Philadelphia, 2007–2011^a^

Characteristic	Females				Males			
	2007, % (SE)	2009, % (SE)	2011, % (SE)	**F_2, 101 _(*P*)**	2007, % (SE)	2009, % (SE)	2011, % (SE)	**F_2, 101 _(*P*)**
Overweight	18.3 (0.01)	21.8 (0.02)	22.3 (0.02)	2.74 (.07)	18.0 (0.01)	16.5 (0.02)	14.2 (0.01)	1.92 (.15)
Obese	14.5 (0.01)	14.2 (0.02)	13.9 (0.01)	0.06 (.94)	17.2 (0.01)	19.4 (0.02)	19.4 (0.02)	0.64 (.53)
Inaccurate weight perception	63.9 (0.03)	59.0 (0.03)	62.1 (0.03)	1.26 (.29)	52.7 (0.03)	56.8 (0.03)	53.9 (0.03)	0.89 (.41)
Trying to lose weight	50.6 (0.02)	54.8 (0.02)	52.2 (0.02)	0.97 (.38)	31.2 (0.02)	33.6 (0.03)	29.6 (.03)	1.2 (.31)
Consumes ≥1 soda per day	31.1^b^ (0.02)	25.5^b^^, ^ c (0.02)	22.5^c^ (0.02)	7.0 (.001)	29.3 (0.02)	29.2 (0.03)	24.2 (0.02)	1.94 (.15)
Consumes ≥5 servings of fruit and vegetables per day	17.0 (0.01)	15.5 (0.02)	18.7 (0.02)	0.83 (.44)	18.6 (0.01)	19.3 (0.02)	19.7 (0.02)	0.09 (.91)
Performs ≥5 days of physical activity per week	26.0^b^ (0.01)	23.6^b^ (0.02)	31.9^c^ (0.02)	6.14 (.003)	37.5 (0.02)	41.4 (0.03)	40.0 (0.02)	0.95 (.39)
Spends ≥2 hours of screen time per day	72.9 (0.01)	73.9 (0.02)	69.1 (0.02)	2.01 (.14)	67.8^b^ (0.02)	77.0^c^ (0.02)	68.2^b^ (0.02)	6.24 (.003)
**Extreme weight-management behavior**								
Any extreme weight-management behavior	19.7 (0.01)	22.4 (0.02)	23.6 (0.02)	1.98 (.14)	16.9 (0.01)	14.8 (0.03)	19.3 (0.02)	1.06 (.35)
Fasting	12.2^b^ (0.01)	13.1^b^^, ^ c (0.02)	17.0^c^ (0.02)	4.10 (.02)	9.2 (0.01)	6.9 (0.01)	11.2 (0.01)	2.08 (.13)
Diet pills	4.8 (0.01)	6.6 (0.01)	6.2 (0.01)	1.05 (.35)	4.0 (0.01)	4.8 (0.01)	3.3 (0.01)	0.41 (.67)
Vomiting	4.7 (0.01)	7.0 (0.01)	5.0 (0.01)	1.09 (.34)	5.5 (0.01)	4.8 (0.01)	3.1 (0.02)	2.05 (.13)

a Models adjusted for students’ age, race/ethnicity, and complex sampling strategy

b, c Within this row, percentages that share a common superscripted letter (b or c) are not statistically different at an α level of .05. Percentages that do not share a common superscripted letter are statistically different.

Among male students, we found no significant differences in the prevalence of overweight or obesity during the study period (Table 2). The percentage of male students reporting 2 or more hours of screen time per day increased significantly from 67.8% in 2007 to 77.0% in 2009 (*P* = .003). We observed no other significant differences in weight-management behaviors or weight perceptions among male students.

In 2011, the final survey year, 79.0% of overweight or obese female students and 63.9% of overweight or obese male students expressed a desire to lose weight, compared with 25.2% of normal-weight female students and 15.1% of normal-weight male students. Among overweight or obese students who wanted to lose weight, we found a significant increase in physical activity and a significant decrease in fruit and vegetable consumption (Table 3) among female students but no significant changes in-weight management behaviors among male students. Among these students in 2011, approximately one-fifth (18.6% of females, 23.3% males) reported having at least 1soda per day, and three-quarters (71.9% of females, 73.4% males) participated in 2 or more hours of screen time each day. Almost one-third of students engaged in 5 or more days of physical activity in the previous week (32.6% of females, 32.8% males) or had 5 servings of fruits and vegetables each day (17.2% of females, 16.6% males). One-third of female students (32.7%) and one-fifth of male students (21.7%) reported using extreme weight-management behaviors.

**Table 3:** Prevalence of Inaccurate Weight Perception and Use of Weight- Management Behaviors Among Overweight and Obese Female and Male Students Who Report Trying to Lose Weight, Philadelphia, 2007–2011^a^

Characteristic	Females				Males			
	2007, % (SE)	2009, % (SE)	2011, % (SE)	**F_2 _(*P*)**	2007, % (SE)	2009, % (SE)	2011, % (SE)	**F_2 _(*P*)**
Inaccurate weight perception	48.3 (0.03)	43.0 (0.05)	47.0 (0.05)	0.5 (.61)	34.9 (0.04)	28.5 (0.05)	31.3 (0.04)	0.7 (.50)
Consumes ≥1 soda per day	23.7 (0.02)	19.7 (0.03)	18.6 (0.03)	1.08 (.34)	26.4 (0.03)	27.5 (0.05)	23.3 (0.04)	0.24 (.79)
Consumes ≥5 servings of fruits and vegetables per day	19.5^b^ (0.02)	10.4^c^ (0.02)	17.2^b^^, ^ c (0.03)	4.03 (.02)	22.7 (0.03)	17.1 (0.03)	16.6 (0.04)	0.9 (.40)
Performs ≥5 days of physical activity per week	23.3^b^ (0.02)	22.9^b^^, ^ c (0.04)	32.6^c^ (0.03)	3.69 (.03)	26.1 (0.04)	36.3 (0.06)	32.8 (0.05)	1.14 (.33)
Spends ≥2 hours of screen time per day	75.6 (0.03)	75.1 (0.03)	71.9 (0.04)	0.4 (.67)	75.9 (0.03)	80.1 (0.04)	73.4 (0.04)	0.84 (.43)
**Extreme weight-management behavior**								
Any extreme weight-management behavior	28.4 (0.03)	32.5 (0.04)	32.7 (0.04)	0.81 (.45)	23.9 (0.03)	17.3 (0.04)	21.7 (0.04)	0.91 (.41)
Fasting	16.4 (0.02)	18.6 (0.03)	24.4 (0.03)	2.7 (.07)	15.7 (0.03)	10.4 (0.03)	16.6 (0.04)	1.62 (.20)
Diet pills	9.4 (0.02)	15.9 (0.04)	11.1 (0.02)	1.62 (.20)	2.7 (0.01)	8.6 (0.04)	3.0 (0.02)	1.37 (.26)
Vomiting	7.9 (0.02)	9.8 (0.03)	7.8 (0.02)	0.24 (.79)	9.1 (0.03)	5.9 (0.02)	2.1 (0.02)	3.10 (.05)

a Models adjusted for students’ age and race/ethnicity.

b, c Within this row, percentages that share a common superscripted letter (b or c) are not statistically different at an α level of .05. Percentages that do not share a common superscripted letter are statistically different.

## Discussion

The current study examined trends in weight status and weight-management behaviors using a representative sample of high school students in Philadelphia. Among these students, we found no significant differences in the prevalence of overweight or obesity according to self-reported height and weight. These findings align with those of another study (3), which found no significant changes in the prevalence of obesity among high school students in Philadelphia and used measured height and weight. Although these trends are encouraging because they may represent a slowing of the youth obesity epidemic, the continued and consistent high prevalence of overweight and obesity among male and female students is a public health problem. Even with a leveling of obesity rates, an estimated 30% of boys and 40% of girls born in the year 2000 are expected to develop diabetes in their lifetime (13). The burden of childhood obesity across the lifespan — for the individual in terms of physical and psychological comorbid conditions (14) and for society in terms of elevated health care costs (15) — is considerable. Therefore, reducing youth overweight and obesity rates remains a public health priority.

The weight-management behaviors of a diverse sample of urban youth reported here are sources of concern. Although we observed improvements in the consumption of sugar-sweetened beverages and participation in regular physical activity among female students, levels of participation in many behaviors important for healthful weight management are still low. Similarly, of the obese and overweight students who reported trying to lose weight, most reported high levels of screen time and low levels of regular physical activity and adequate fruit and vegetable consumption. Of special concern, one-quarter of overweight or obese students who reported trying to lose weight used extreme weight-management behaviors such as fasting, diet pills, and vomiting. Together, these data point to a myriad of poor weight-management behaviors that could belie the stabilizing levels of BMI and suggest that public health efforts do not provide adolescents with helpful information on how to address their weight healthfully.

Our data showed that at least one-half of female students and approximately one-third of male students reported trying to lose weight. Evidence of such a motivated population could be interpreted as a public health opportunity to replace ineffective weight-management efforts with more healthful behaviors. Population-level efforts that address the individual, social, and environmental determinants of weight- management behaviors in conjunction with public policy initiatives (16,17) may have contributed to the stabilization of youth obesity rates (2). Continued progress may require more intense programming efforts so that healthful weight-loss behaviors are adopted instead of extreme behaviors, particularly in metropolitan areas such as Philadelphia that are characterized by environments that do not offer ready access to healthful foods (18) or adequate and safe facilities for physical activity (19). Another population-level barrier to the promotion of healthful weight-loss behaviors is the reticence of health care professionals to address childhood obesity and prescribe behavior-management strategies for this high-risk population (20,21); thus, a concerted effort is needed to increase the efficacy of health care professionals to address childhood obesity and promote increased physical activity and healthful diets.

Our study had several limitations, including the use of self-reported data instead of objective measures. Additionally, because of the wide range of behaviors assessed by the YRBS during a single class period, we largely used single-item measures to examine weight-management behaviors. Use of single-item measures may have affected the validity and reliability of the study findings. Despite these limitations, data obtained by the YRBS are essential for understanding the behavioral patterns of adolescents in Philadelphia and the United States as a whole, and findings can serve as a basis for further analytical research.

Findings from this descriptive study and other recent studies that show a plateau in the prevalence of overweight and obesity among high school students (3) suggest that continued vigilance in educating high school students about healthful dietary and physical activity practices and promotion of environments that support healthful weight-related behaviors are necessary. Additionally, given the tenacity of overweight and obesity rates among high school students (3), continued research is needed to identify policies and programs that resonate with this age group. Finally, greater access to targeted community-based obesity prevention and treatment programs is needed for the large proportion of young people who desire to achieve a healthful weight; these programs are especially needed because of the propensity of high school students to engage in unhealthy behaviors, such extreme weight-management behaviors and extensive screen time, while trying to lose weight.

## Appendix: Variables Examined in Study on Obesity and Weight-Related Behaviors Among High School Students in Philadelphia, Pennsylvania, and Questions Used From Youth Risk Behavior Survey, 2007, 2009, and 2011

**1. Weight perception**

How do you describe your weight?

- Very underweight
- Slightly underweight
- About the right weight
- Slightly overweight
- Very overweight

**2. Weight-loss intention (yes/no)**

Which of the following are you trying to do about your weight?

- Lose weight
- Gain weight
- Stay the same weight
- I am not trying to do anything about my weight

Participants who chose option a were coded as intending to lose weight.

**3. Soda consumption (≥1 per day in last week)**

During the past 7 days, how many times did you drink a can, bottle, or glass of soda or pop, such as Coke, Pepsi, or Sprite? (Do not count diet soda or diet pop.)

- I did not drink soda or pop during the past 7 days
- 1 to 3 times during the past 7 days
- 4 to 6 times during the past 7 days
- 1 time per day
- 2 times per day
- 3 times per day
- 4 or more times per day

Participants who chose any of options d through g were coded as having 1 or more soda drinks in the last week.

**4. Fruit and vegetable consumption (≥5 servings per day in the last week)**

Composite variable calculated from responses to the following questions:

During the past 7 days, how many times did you drink 100% fruit juices such as orange juice, apple juice, or grape juice? (Do not count punch, Kool-Aid, sports drinks, or other fruit-flavored drinks.)

- I did not drink 100% fruit juice during the past 7 days
- 1 to 3 times during the past 7 days
- 4 to 6 times during the past 7 days
- 1 time per day
- 2 times per day
- 3 times per day
- 4 or more times per day

During the past 7 days, how many times did you eat fruit? (Do not count fruit juice.)

- I did not eat fruit during the past 7 days
- 1 to 3 times during the past 7 days
- 4 to 6 times during the past 7 days
- 1 time per day
- 2 times per day
- 3 times per day
- 4 or more times per day

During the past 7 days, how many times did you eat green salad?

- I did not eat green salad during the past 7 days
- 1 to 3 times during the past 7 days
- 4 to 6 times during the past 7 days
- 1 time per day
- 2 times per day
- 3 times per day
- 4 or more times per day

During the past 7 days, how many times did you eat potatoes? (Do not count french fries, fried potatoes, or potato chips.)

- I did not eat potatoes during the past 7 days
- 1 to 3 times during the past 7 days
- 4 to 6 times during the past 7 days
- 1 time per day
- 2 times per day
- 3 times per day
- 4 or more times per day

During the past 7 days, how many times did you eat carrots?

- I did not eat carrots during the past 7 days
- 1 to 3 times during the past 7 days
- 4 to 6 times during the past 7 days
- 1 time per day
- 2 times per day
- 3 times per day
- 4 or more times per day

During the past 7 days, how many times did you eat other vegetables? (Do not count green salad, potatoes, or carrots.)

- I did not eat other vegetables during the past 7 days
- 1 to 3 times during the past 7 days
- 4 to 6 times during the past 7 days
- 1 time per day
- 2 times per day
- 3 times per day
- 4 or more times per day

Variable computed as directed in the 2011 Youth Risk Behavior Survey Data User’s Guide (p. 45). For each contributing question, a was assigned a value of 0, b = 2/7, c = 5/7, d = 1, e = 2, f = 4 and g = 4. Responses were totaled across each contributing question to achieve the total daily servings of fruits and vegetables during the past 7 days.

**5. Physical activity levels (active for ≥60 minutes per day on 5 of the last 7 days)**

During the past 7 days, on how many days were you physically active for a total of at least 60 minutes per day? (Add up all the time you spent in any kind of physical activity that increased your heart rate and made you breathe hard some of the time.)

- 0 days
- 1 day
- 2 days
- 3 days
- 4 days
- 5 days
- 6 days
- 7 days

Participants who chose any of options f, g, or h were coded as being active for at least 60 minutes per day on 5 of the last 7 days.

**6. Television viewing**

On an average school day, how many hours do you watch TV?

- I do not watch TV on an average school day
- Less than 1 hour per day
- 1 hour per day
- 2 hours per day
- 3 hours per day
- 4 hours per day
- 5 or more hours per day

**7. Recreational computer use**

On an average school day, how many hours do you play video or computer games or use a computer for something that is not school work? (Include activities such as Xbox, PlayStation, Nintendo DS, iPod touch, Facebook, and the Internet.)

- I do not play video or computer games or use a computer for something that is not school work
- Less than 1 hour per day
- 1 hour per day
- 2 hours per day
- 3 hours per day
- 4 hours per day
- 5 or more hours per day

**8. Extreme weight-management strategies**

During the past 30 days, did you go without eating for 24 hours or more (also called fasting) to lose weight or to keep from gaining weight?

- Yes
- No

During the past 30 days, did you take any diet pills, powders, or liquids without a doctor’s advice to lose weight or to keep from gaining weight? (Do not include meal replacement products such as Slim Fast.)

- Yes
- No

During the past 30 days, did you vomit or take laxatives to lose weight or to keep from gaining weight?

- Yes
- No
